# Posterior Tibial Neurectomy

**Published:** 1898-08

**Authors:** 


POSTERIOR TIBIAL NEURECTOMY may be resorted to in ringbone when other methods have failed. In one case the lameness of spavin yielded to this operation after having been subjected to all forms of treatment during two years.

